# Identification of risk factors for high-risk dedifferentiation in papillary thyroid carcinoma and construction of discriminative model

**DOI:** 10.3389/fonc.2025.1535966

**Published:** 2025-06-04

**Authors:** Xiang Liu, Qiao-li Zhu, Zi-yi He, Jing-de Shu, Cheng Xiang

**Affiliations:** ^1^ Department of Breast and Thyroid Surgery, The Quzhou Affiliated Hospital of Wenzhou Medical University, Quzhou People’s Hospital, Quzhou, Zhejiang, China; ^2^ Department of Thyroid Surgery, The Second Affiliated Hospital of Zhejiang University College of Medicine, Hangzhou, Zhejiang, China

**Keywords:** thyroid differentiation score, papillary thyroid carcinoma, dedifferentiation, differentially expressed genes, CD55

## Abstract

**Objective:**

We initially found that the thyroid differentiation score (TDS) was associated with the prognosis of papillary thyroid carcinoma (PTC) patients. Therefore, this study aimed to investigate the influencing factors and construct a discriminative model of high-risk dedifferentiation, and to explore the possible mechanisms.

**Methods:**

Data were sourced from the TCGA database. The influences of the TDS, tumor mutation burden, and immune score on the progression-free interval (PFI) were assessed by the Kaplan-Meier method and multivariable Cox regression. Then, logistic regression analyses were utilized to explore the factors of dedifferentiation and a nomogram model was conducted. Additionally, differentially expressed genes (DEGs) were identified using *RNA* sequencing data, while their regulatory pathways were determined by the Kyoto Encyclopedia of Genes and Genomes (KEGG) analysis. Finally, the differential expression of key genes of major pathways was explored.

**Results:**

This study included 391 PTC patients. After analyzing the influences of the three indicators on survival, only TDS showed an association with PFI. Multivariable logistic analysis revealed that the disease duration and PTC subtypes influenced dedifferentiation. The nomogram model based on these two variables showed improved discriminative capability. The study identified 17 overlapping DEGs associated with the dedifferentiation and three primary enrichment pathways, with complement and coagulation cascade pathways being the most significant (*P*<0.001). The central gene was *CD55*, which showed high expression in high-risk dedifferentiated and tall cell PTC, and the expression level increased as the disease progressed.

**Conclusion:**

This research may contribute to promising identifying high-risk dedifferentiated PTC and also provide a potential therapeutic target.

## 1 Introduction

Thyroid cancer (THCA) is the most prevalent endocrine system malignancy, with 821,000 new cases worldwide in 2022, accounting for 4.1% of all new cancer cases globally and ranking 7th among malignant tumors (1, 2). Papillary thyroid carcinoma (PTC) is the most common subtype of THCA, accounting for approximately 80%-90% of all cases (3). PTC originates from follicular epithelium (4). It includes more than ten subtypes, such as classical/usual PTC, infiltrating follicular PTC, tall cell PTC (TCPTC), and columnar cell PTC (5). PTC is highly differentiated and prone to regional lymph node metastasis at an early stage, with low local infiltration and recurrence rate, resulting in a better prognosis. The 5-year survival rates of PTC range from 83% to 98%, while well-differentiated classic PTC has a 10-year survival rate of up to 97% (6, 7). However, some PTC subtypes may undergo dedifferentiation, making the tumor cells more aggressive and losing iodide uptake capacity, then leading to increased disease progression and mortality (8–10). Compared to identifying dedifferentiation through histopathology alone, the thyroid differentiation score (TDS) integrates the *mRNA* expression levels of 16 genes associated with thyroid metabolism and function, providing a more consistent method for identifying dedifferentiation (11, 12).

With the advancements in immuno-oncology, immune checkpoint inhibitor (ICI) therapies have revolutionized cancer treatment. However, these groundbreaking therapies also have side effects (13). Tumor mutation burden (TMB), as an emerging biomarker, is considered to be a promising predictor of response to ICI therapy (14). Research has demonstrated that elevated TMB is associated with the response to ICI in several types of tumors. For example, in non-small cell lung cancer, high TMB is linked to significantly improved progression-free interval (PFI) in patients receiving combined Nivolumab and Ipilimumab treatment (15). Similarly, increased TMB is associated with improved survival rates in head and neck cancer and bladder cancer patients undergoing ICI therapy (16).

The tumor immune microenvironment (TIME) plays a crucial role in cancer progression and influences treatment outcomes and prognosis (17). In TIME, the key cluster cells that are most likely to influence clinical outcomes of THCA may be immune cells (18). Currently, immune scores can be obtained using multiple computer algorithms such as Cibersort, Timer, and ImmuCellAI, which can assess immune cell infiltration by *RNA-seq* expression data (19–21). PTC immune infiltrating cells such as dendritic cells, biased M2 phenotype tumor-associated macrophages, and mast cells are associated with tumor differentiation or anti-tumor immune responses (22–24). Several studies have investigated the relationship between known differentially expressed genes (DEGs) in PTC, TIME, and prognosis (25–27). However, these studies did not include currently unproven immune-related genes or prognostic genes that were not differentially expressed in the analysis. In addition, models that incorporate multiple genes limit the feasibility of their clinical application. To address these limitations, this study employed the ESTIMATE algorithm to calculate immune scores for PTC cases.

Dedifferentiation, gene mutation, and immune microenvironment significantly impact the biological behavior and clinical prognosis of PTC. In this study, we initially explored the effects of these three markers on PFI in PTC patients, revealing that only TDS influenced prognosis. Subsequently, the influencing factors and mechanisms were explored for TDS to guide intervention strategies.

## 2 Materials and methods

### 2.1 Data acquisition and preprocessing

The transcriptome data and clinical information data of the PTC patients were from the Cancer Genome Atlas (TCGA) genome database (https://portal.gdc.cancer.gov/). PTC samples in the TCGA database were collected and sequenced primarily between 2010 and 2015. We obtained 507 PTC patients’ data, and 391 of them with complete TDS, TMB, and immune scores data were analyzed. Perl version 5.24.3 software was utilized to transform original *RNA* sequencing data into an RNA expression matrix.

### 2.2 Calculation of immune score, TDS, and TMB in PTC patients

ESTIMATE, a computerized algorithm, can infer the level of stromal and immune cell infiltration in tumor tissue based on expression profiles (28). The immune score of the immune microenvironment in PTC patients was calculated by the ESTIMATE algorithm.

TDS encompasses the expression levels of 16 thyroid metabolic and functional *mRNAs* (12). The log_2_ normalized RSEM values were first centered on the median of each sample to derive the log_2_ (fold-change) (FC), and then the TDS of each tumor tissue was obtained by summing the 16 genes of each sample. The calculation formula was as follows: TDS = log_2_ (FC) average of 16 genes (29, 30). The genes involved were *DIO1*, *DIO2*, *DUOX1*, *DUOX2*, *FOXE1*, *GLIS3*, *NKX2-1*, *PAX8*, *SLC26A4*, *SLC5A5*, *SLC5A8*, *TG*, *THRA*, *THRB*, *TPO*, and *TSHR*.

TMB was defined as the number of somatic, coding, base substitution, and insertion-deletion variants per megabase (Mb) of the examined target genomic region (14). The formula was calculated as follows: sample TMB = number of mutations/exon region size (31). The TMB distribution for patients was directly acquired from the TCGA database.

### 2.3 Key indicators identification

We first explored the influence of immune score, TDS, and TMB on the survival outcome of PTC patients by Kaplan-Meier (KM) method and log-rank test. Due to the long overall survival and tumor-specific survival of PTC patients, this study collected limited mortality data. PFI was used as the survival indicator. PFI is defined as the time interval between the date of diagnosis of the disease and the occurrence of a new tumor event, including disease progression, local recurrence, distant metastasis, the new primary tumor, or death from a tumor (32). In the KM analysis, all the patients were divided into high and low groups according to the optimal cut-off value and median of TDS, TMB, and immune scores. The optimal cut-off value is the minimum *P*-value cut-off for univariable Cox analysis when PFI is the primary endpoint and is obtained from the R software package “survminer”. We then performed a Cox regression analysis to assess their association with PFI. The factor of PFI was regarded as the key indicator.

### 2.4 Definition of high/low-risk dedifferentiation

In this study, only TDS showed an association with PFI, thus becoming the research topic in the subsequent analysis. The 391 PTC patients were grouped by the optimal cut-off value (-0.303) of the TDS score. The low-differentiation score group (TDS≤-0.303) was defined as high-risk dedifferentiation, while the high-differentiation score group (TDS>-0.303) was designated as low-risk dedifferentiation. We then compared the baseline data between high and low-risk dedifferentiation groups.

### 2.5 Collected baseline variables

Relevant variables collected in this study were related to: demographic characteristics (age, sex, race), tumor-related clinical variables (disease duration, tumor size, TNM stage, PTC subtypes, stage, site, and focus type of primary lesions, lymph node preoperative assessment diagnostic imaging type, medical history of the thyroid gland disorder, radiation therapy and response to therapy, postoperative tumor residue after resection) and follow-up (follow-up after radiation treatment, PTC status after initial treatment).

The disease duration was defined as the time interval between initial diagnosis and completion of the TCGA program. Tumors with ≥99% of follicular structures were considered to be follicular variant PTC, and those with tall cells content ≥50% were defined as TCPTC. It has been noted that stage 1 and 2 tumors remain confined to the thyroid gland and have not yet spread to the central compartment of the lymph nodes; in stages 3 and 4, the cancer spreads to the lymph nodes, including other organs (33). Therefore, we combined stage 1 and 2 samples as early-stage samples and stage 3 and 4 samples as advanced-stage samples. Preoperative lymph node imaging was categorized as ultrasound-only or other. The other included computed tomography (CT)-only, magnetic resonance imaging (MRI)-only, or combinations. Response to radiotherapy was classified as complete response or other, with other conditions including partial response, stable disease, and radiographic progressive disease. Residual tumors after resection were categorized as absent or present. Complete resection of the tumor was defined as no tumor residue, resection with residue under a microscope and residue visible to the naked eye was classified as having residue. In the follow-up results, the status of PTC after initial treatment included both no imaging evidence of disease and disease persistence. Disease persistence included persistent locoregional disease and persistent distant metastases. In spite some samples had missing data, which are clinically valuable and worth analyzing, they were retained for baseline and/or subsequent analyses.

### 2.6 Influencing factors and model construction of high-risk dedifferentiation

After baseline comparison, we conducted a logistic regression analysis to explore the key factors and established a nomogram model for predicting the high-risk dedifferentiation. The performance of the nomogram model was then evaluated by several analyses. The detailed information is shown in the following Statistical analysis section.

### 2.7 Potential mechanism exploration associated with dedifferentiation

The *RNA-seq* expression data of PTC patients were normalized using the log_2_ (X+1) method. **Supplementary Table S1** displays the normalized data of 16 TDS-related genes and CD55. The “Limma” package in R software was used to detect the differentially expressed genes (DEGs) between the high-risk dedifferentiation group and the low-risk differentiation group (DEG1), as well as high and low nomogram score groups (DEG2), based on the threshold of adjusted *P*-value < 0.05 and |log_2_ (FC)| > 1. Gene expression volcano plots were created with Graphpad Prism 8. Overlapping genes between DEG1 and DEG2 were identified using Venn diagrams (bioinformatics.psb.ugent.be/webtools/Venn/). Kyoto Encyclopedia of Genes and Genomes (KEGG) enrichment analysis was performed to determine the pathways associated with the overlapping genes and identify genes in major pathways. Finally, the expression levels of key genes involved in the major pathways were analyzed for differences between PTC subtypes and dedifferentiation risk groups, and the correlation between gene expression level and disease duration was also assessed.

### 2.8 Statistical analysis

Continuous variables with non-normal distribution were characterized by medians and quartiles (P25, P75), and the differences between high and low-risk dedifferentiation groups were compared using the Wilcoxon-Mann-Whitney test. Categorical variables were presented as frequencies (n) and proportions (%) and then compared by the Chi-square test/Fisher exact test. Variables that were statistically different from baseline comparisons were included in logistic regression analyses to explore the factors influencing high-risk dedifferentiation in PTC patients. Collinearity analysis was performed due to the joint confirmation of tumor staging by T, N, and M staging indicators, and the potential for interactions among other indicators. Collinearity was considered present when the variance inflation factor (VIF) exceeded 10.

Based on the results of multivariable logistic regression, a model for high-risk dedifferentiation was constructed using the nomogram method. Receiver operating characteristic analysis (ROC) was used to evaluate the performance of the key factors in predicting high-risk dedifferentiation using “pROC” packages, while decision curve analysis (DCA) based on “rmda” packages was used to assess the net clinical benefit of the model. In addition, based on the “dplyr” and “Hmisc” packages in the R software, integrated discrimination improvement (IDI) analysis and net reclassification improvement (NRI) analysis were conducted to explore the improvement in the performance of the models compared to the individual influences. Restricted cubic spline (RCS) analysis via “rms” packages was utilized to explore the association of nomogram score and dedifferentiation. These analyses were performed using R software (version 4.4.1), with statistical significance set at *P* < 0.05.

## 3 Results

### 3.1 The influence of three biomarkers on PFI

**Figure 1:**
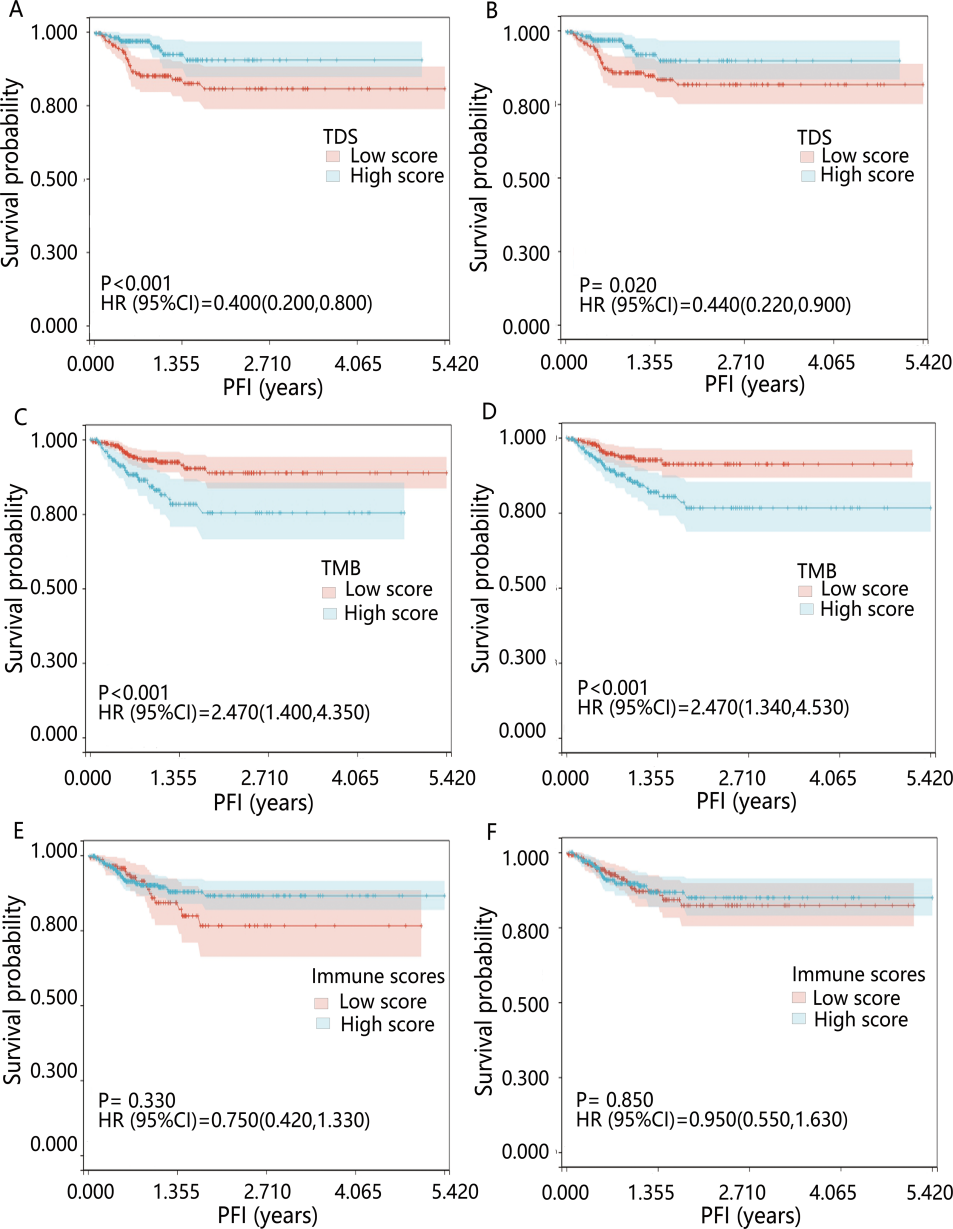
Kaplan-Meier survival curves for PFI across various biomarkers. Patients were grouped by **(A)** optimal cut-off value of TDS; **(B)** median TDS; **(C)** optimal cut-off value of TMB; **(D)** median TMB; **(E)** optimal cut-off value of immune scores; **(F)** median immune scores. HR was derived from the results of the univariable Cox regression analysis.

The influence of three tumor biomarkers including TDS, TMB, and immune scores on PFI was analyzed in 391 PTC patients. All patients were divided into high and low-score groups according to the optimal cut-off value and median of TDS, TMB, and immune scores. For the TDS score, the high-differentiated group had a better prognosis compared to the low-differentiated group (*P*<0.05) (**Figures 1A, B**). Regarding TMB, groups with high mutation burden showed worse prognosis compared to the low mutation burden group (*P*<0.001) (**Figures 1C, D**). Immune score showed no significant influence on prognosis (*P*>0.05) (**Figures 1E, F**). The results of multivariable Cox regression analysis revealed that on both continuous and dichotomous TDS, TMB, and immune scores categorized by the optimal cut-off value, only TDS remained an influencing factor of PFI after adjusted age, sex, and race (**Table 1**). Due to the association between TDS and PFI, therefore the differentiation status of PTC patients became the topic of our next analysis.

**Table 1:** Results of multivariable Cox analysis of the influences of TDS, TMB and immune scores on PFI in PTC patients.

Variables	HR	95%CI	*P*-value
COX model 1			
Age	1.018	0.997-1.039	0.093
Gender	0.770	0.372-1.594	0.481
Race	0.644	0.190-2.183	0.480
TDS as continuous variable	0.691	0.506-0.945	0.021
TMB as continuous variable	1.285	0.875-1.888	0.201
Immune score as continuous variable	1.000	1.000-1.000	0.179
COX model 2			
Age	1.012	0.989,1.035	0.314
Gender	0.763	0.370,1.573	0.463
Race	0.636	0.191,2.117	0.461
TDS as dichotomized variable	2.480	1.186,5.185	0.016
TMB as dichotomized variable	1.615	0.779,3.344	0.197
Immune score as dichotomized variable	0.533	0.264,1.076	0.079

HR, Hazard ratio; CI, Confidence interval; TDS, Thyroid differentiation score; TMB, Tumor mutation burden.

### 3.2 Baseline characteristics of high/low-risk dedifferentiation groups

**Table 2** demonstrates the differences in baseline data between the high/low-risk dedifferentiation groups. The median age of 391 PTC patients was 46.000 (34.000,58.000). The majority were non-Hispanic (90.064%), with a lower proportion in the high-risk group compared to the low-risk group (86.250% vs. 94.079%, *P*=0.021). Among the continuous variables, disease duration, tumor length, tumor width, number of examined lymph nodes, and number of positive lymph nodes were higher in the high-risk group than the low-risk group (all *P*<0.05). Among the categorical variables, 28.302% of patients in the high-risk group had a medical history of thyroid gland disorder, lower than the low-risk group (44.643%) (*P*=0.006). The percentage of patients with classic/usual PTC was higher in the high-risk group than in the low-risk group (82.065% vs 60.099%) (*P*<0.001). T3 stage, T4 stage, N1 stage, advanced stage, follow-up after radiation treatment, and residual tumor were more prevalent in the high-risk group than in the low-risk group (all *P*<0.05).

**Table 2:** The baseline information of participants grouped by the risk of dedifferentiation.

Variables	Total (n=391)	Low risk (n=205)	High risk (n=186)	*P*-value
Age, years	391(100.000)	46.000(34.000,58.000)	47.000(34.000,58.000)	0.904
Tumor depth, cm	319(81.586)	1.500(1.000,2.000)	1.600(1.000,2.500)	0.240
Tumor length, cm	369(94.373)	2.400(1.500,3.500)	2.800(1.800,4.000)	0.016
Tumor width, cm	334(85.422)	1.900(1.200,2.700)	2.200(1.500,3.000)	0.015
Number of examined lymph nodes	297(75.959)	4.000(2.000,10.000)	7.000(3.000,17.000)	0.014
Number of positive lymph nodes	294(75.192)	0.000(0.000,3.000)	2.000(0.000,6.000)	<0.001
I-131 doses, mCi	184(47.059)	100.000(75.200,125.000)	101.000(94.600,150.000)	0.268
Disease duration, years	390(99.744)	1.000(1.000,3.000)	2.000(1.000,5.000)	<0.001
Gender, n(%)				0.715
Male	100(25.575)	54(26.341)	46(24.731)	
Female	291(74.425)	151(73.659)	140(75.269)	
Race, n(%)				0.021
Non-Hispanic	281(90.064)	143(94.079)	138(86.250)	
Hispanic	31(9.936)	9(5.921)	22(13.750)	
PTC subtypes, n(%)				<0.001
Classical/usual	273(70.543)	122(60.099)	151(82.065)	
Follicular	85(21.964)	76(37.438)	9(4.891)	
Tall cell	29(7.494)	5(2.463)	24(13.043)	
Stage, n(%)				0.002
Early	268(68.895)	155(75.980)	113(61.081)	
Advanced	121(31.105)	49(24.020)	72(38.919)	
T stage, n(%)				<0.001
T1	114(29.306)	73(35.784)	41(22.162)	
T2	131(33.676)	78(38.235)	53(28.649)	
T3	129(33.162)	52(25.490)	77(41.622)	
T4	15(3.856)	1(0.490)	14(7.568)	
N stage, n(%)				<0.001
N0	186(47.570)	119(58.049)	67(36.022)	
N1	205(52.430)	86(41.951)	119(63.978)	
M stage, n(%)				0.358
M0	217(55.641)	109(53.431)	108(58.065)	
M1	173(44.359)	95(46.569)	78(41.935)	
PTC status after initial treatment, n(%)				0.083
Tumor free	233(91.016)	135(93.750)	98(87.500)	
Persistent	23(8.984)	9(6.250)	14(12.500)	
Follow-up after radiation treatment, n(%)				0.010
No	136(39.306)	84(45.652)	52(32.099)	
Yes	210(60.694)	100(54.348)	110(67.901)	
Lymph node preoperative assessment diagnostic imaging type, n(%)				0.830
Ultrasound	225(78.947)	116(79.452)	109(78.417)	
Other	60(21.053)	30(20.548)	30(21.583)	
Medical history of thyroid gland disorder, n(%)				0.006
Normal	207(63.303)	93(55.357)	114(71.698)	
Nodular hyperplasia	57(17.431)	38(22.619)	19(11.950)	
Lymphocytic thyroiditis	63(19.266)	37(22.024)	26(16.352)	
Primary neoplasm focus type, n(%)				0.569
Unifocal	206(53.927)	104(52.525)	102(55.435)	
Multifocal	176(46.073)	94(47.475)	82(44.565)	
Primary site, n(%)				0.106
Left lobe	140(36.269)	68(33.333)	72(39.560)	
Right lobe	166(43.005)	98(48.039)	68(37.363)	
Bilateral and isthmus	80(20.725)	38(18.627)	42(23.077)	
Radiation, n(%)				0.057
No	55(35.714)	31(43.662)	24(28.916)	
Yes	99(64.286)	40(56.338)	59(71.084)	
Radiation response, n(%)				0.511
Complete	120(85.106)	55(87.302)	65(83.333)	
Other	21(14.894)	8(12.698)	13(16.667)	
Residual tumor, n(%)				0.023
Absent	306(88.696)	169(92.350)	137(84.568)	
Present	39(11.304)	14(7.650)	25(15.432)	

PTC, Papillary thyroid carcinoma.

### 3.3 The influencing factors of high-risk dedifferentiation

**Figure 2:**
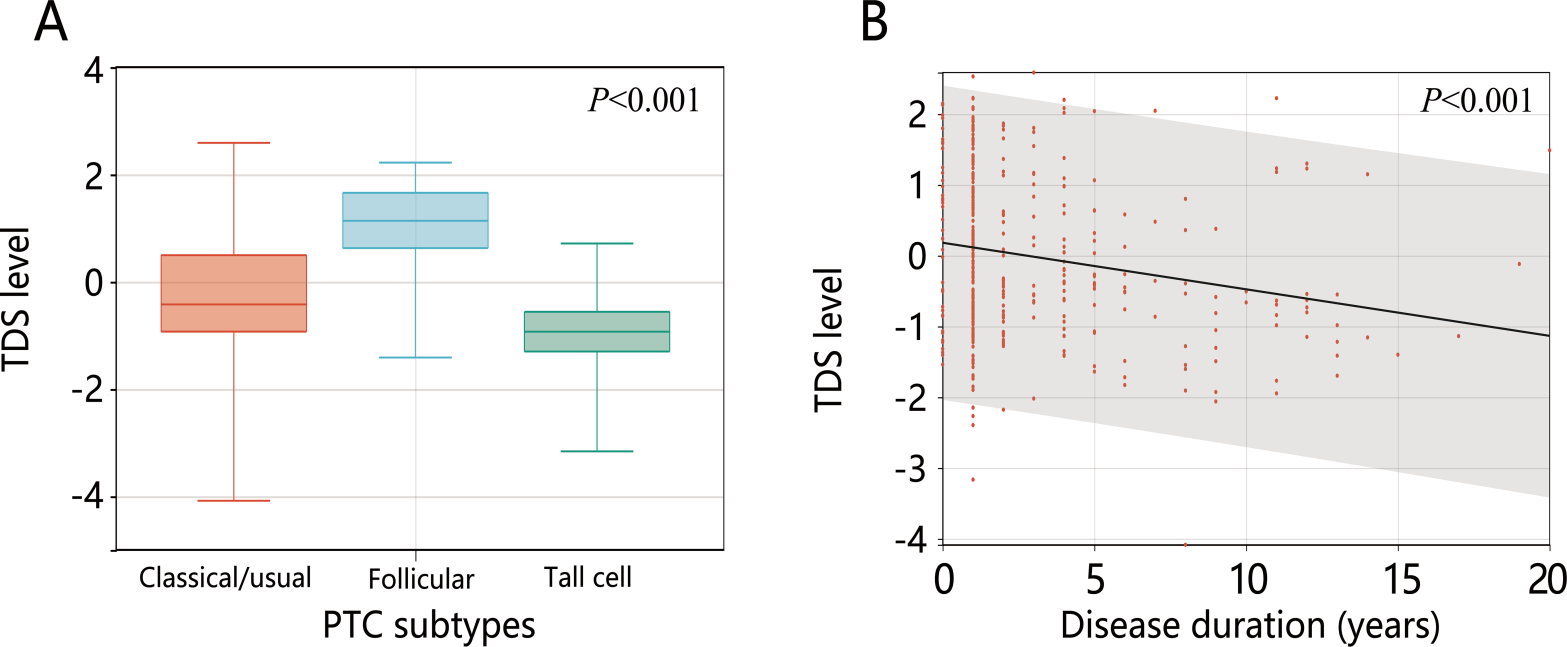
The levels of TDS in **(A)** different PTC subtypes and **(B)** disease duration.

Collinearity analysis of the 13 significantly different variables revealed no collinearity (all VIF<10) (**Supplementary Table S2**). The 13 variables were included in the logistic regression analysis. The results of univariable logistic regression showed that except for the number of examined lymph nodes, the other 12 variables were related to dedifferentiation (all *P*<0.05). Further multivariable logistic analysis containing all variables showed that only disease duration and PTC subtypes were influential factors for high-risk dedifferentiated PTC. Follicular variant PTC was a protective factor for high-risk dedifferentiation (OR=0.207, 95%CI: 0.035-0.872) and tall cell PTC was a risk factor for it (OR=8.035, 95%CI: 1.389-72.04). Longer disease duration increased the likelihood of dedifferentiation (OR=1.192, 95%CI: 1.018-1.427) (all *P*<0.05) (**Table 3**). Further analysis revealed that TCPTC had the lowest TDS, and TDS decreased with increasing disease duration (**Figures 2A, B**).

**Table 3:** Results of logistic regression analysis of factors associating with high-risk of dedifferentiation.

Variables	Univariable analysis	Multivariable analysis
	OR(95%CI)	OR(95%CI)
Tumor length	1.168(1.025-1.332)**	0.752(0.356-1.531)
Tumor width	1.230(1.023-1.477)**	2.131(0.828-5.920)
Number of examined lymph nodes	1.011(0.996-1.027)	0.976(0.932-1.020)
Number of positive lymph nodes	1.048(1.003-1.096)**	1.071(0.940-1.242)
Disease duration, years	1.136(1.065-1.212)***	1.192(1.018-1.427)**
Race		
Non- Hispanic	Reference	Reference
Hispanic	2.533(1.127-5.694)**	2.398(0.614-11.466)
PTC subtypes		
Classical/usual	Reference	Reference
Follicular	0.096(0.046-0.199)***	0.207(0.035-0.872)**
Tall cell	3.878(1.437-10.464)**	8.035(1.389-72.04)**
T stage		
T1	Reference	Reference
T2	1.210(0.721-2.030)	0.592(0.162-2.027)
T3	2.636(1.568-4.433)***	1.511(0.368-6.093)
T4	24.927(3.163-196.456)**	3.94(0.182-146.565)
N stage		
N0	Reference	Reference
N1	2.458(1.634-3.696)***	2.199(0.749-6.585)
Stage		
Early	Reference	Reference
Advanced	2.016(1.303-3.119)**	0.381(0.099-1.309)
Follow-up after radiation treatment		
No	Reference	Reference
Yes	1.777(1.145-2.757)**	0.948(0.346-2.534)
Medical history of thyroid gland disorder		
Normal	Reference	Reference
Nodular hyperplasia	0.408(0.221-0.754)**	1.556(0.394-6.212)
Lymphocytic thyroiditis	0.573(0.324-1.015)	0.819(0.283-2.355)
Residual tumor		
Absent	Reference	Reference
Present	2.203(1.103-4.400)**	2.158(0.403-14.145)

* : *P*<0.05,**; *P*<0.001,***.

OR, Odds ratio; CI, Confidence interval; PTC, Papillary thyroid carcinoma.

### 3.4 Nomogram model construction and performance evaluation

**Figure 3:**
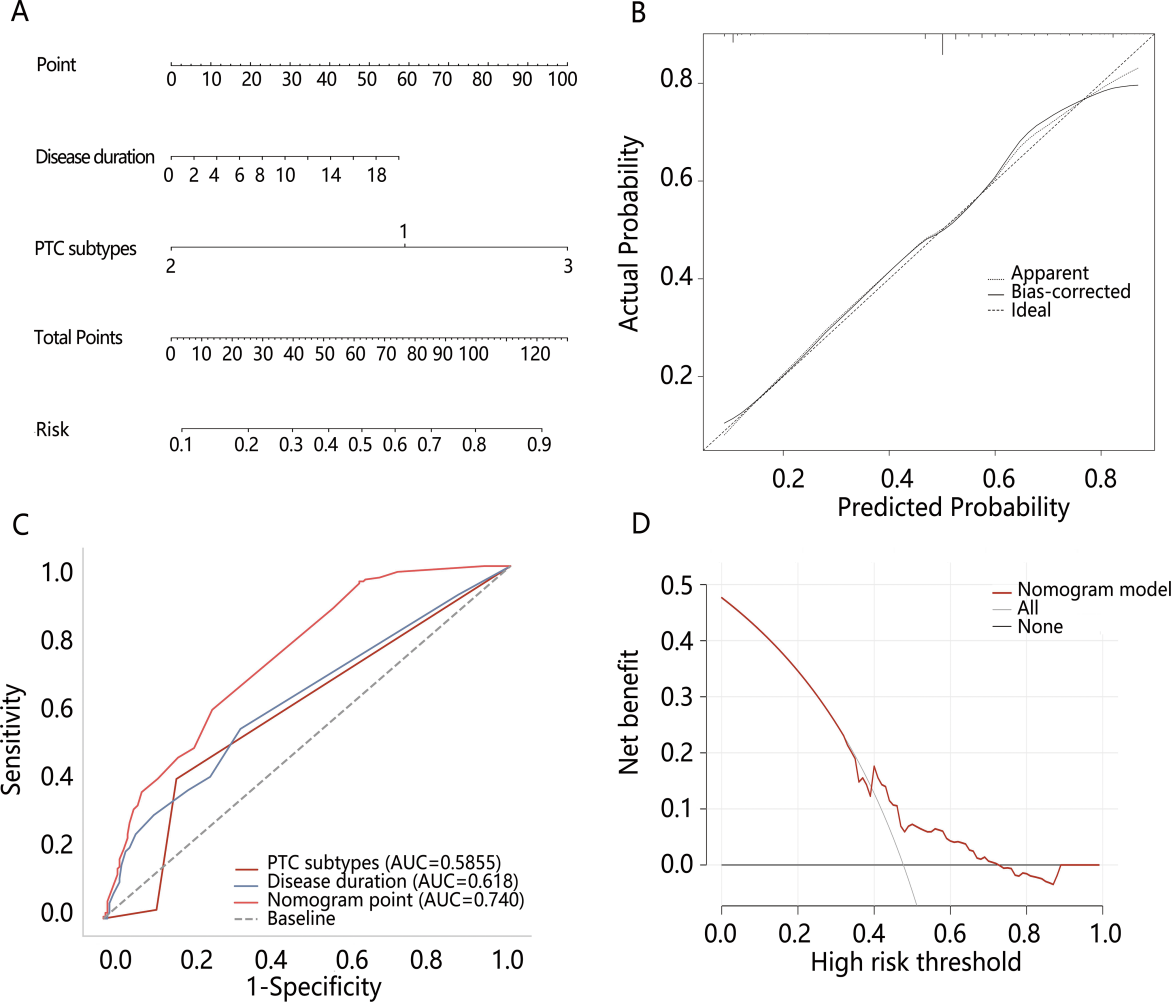
The construction and evaluation of the nomogram model. **(A)** The nomogram model of high-risk dedifferentiation; **(B)** The calibration curve of the nomogram model; **(C)** Comparison of ROC between the nomogram model and different variables; **(D)** The DCA of the nomogram model.

Based on the multivariable logistic regression results, the nomogram model was constructed based on disease duration and PTC subtypes. Meanwhile, the calibration curve of the nomogram model was plotted, demonstrating good calibration (**Figures 3A, B**). The performance of disease duration and PTC subtypes as well as the nomogram model in predicting high-risk dedifferentiation was further evaluated by ROC analysis (**Table 4**, **Figure 3C**). Results indicated that PTC subtypes had the highest specificity (0.821), while the nomogram model exhibited the highest AUC (0.740), sensitivity (0.957), Youden index (0.328), and accuracy (0.524). The Delong test revealed that the AUC value of the nomogram model was significantly higher than the AUC values for disease duration alone and PTC subtypes alone (all *P*<0.001). DCA confirmed that the nomogram model could provide a net clinical benefit, with a risk interval of 0.40-0.76 (**Figure 3D**). IDI analysis showed that the performance of the nomogram model was improved by 15.3% compared to single disease duration (IDI=0.153, 95%CI: 0.118-0.188, *P*<0.001) and 18.8% improvement compared to PTC subtypes (IDI=0.188, 95%CI: 0.149-0.228, *P*<0.001). NRI analysis suggested that nomogram model discriminative performance improved by 25.8% and 5.7% compared to PTC subtypes (NRI=0.258, 95%CI: 0.180-0.337) and disease duration (NRI=0.057, 95%CI: -0.039-0.169), respectively. These results indicated that the constructed nomogram model possessed good discriminative capability.

**Table 4:** The results of ROC analysis of factors associated with high-risk dedifferentiation.

Variables	PTC subtypes	Disease duration	Nomogram Point
AUC	0.585(0.534-0.620)	0.618(0.572-0.667)	0.740(0.696-0.792)
Sensitivity	0.396(0.338-0.458)	0.538(0.239-0.606)	0.957(0.575-0.987)
Specificity	0.821(0.754-0.860)	0.663(0.617-0.927)	0.371(0.323-0.781)
Youden index	0.217(0.133-0.288)	0.201(0.149-0.286)	0.328(0.268-0.426)
Accuracy	0.476(0.410-0.535)	0.523(0.468-0.578)	0.524(0.480-0.574)
Best cutoff	2.000(2.000-2.000)	2.000(2.000-6.000)	37.338(14.361-64.719)
Delong test	*P*<0.001	*P*<0.001	/

### 3.5 Potential mechanism associated with dedifferentiation

**Figure 4:**
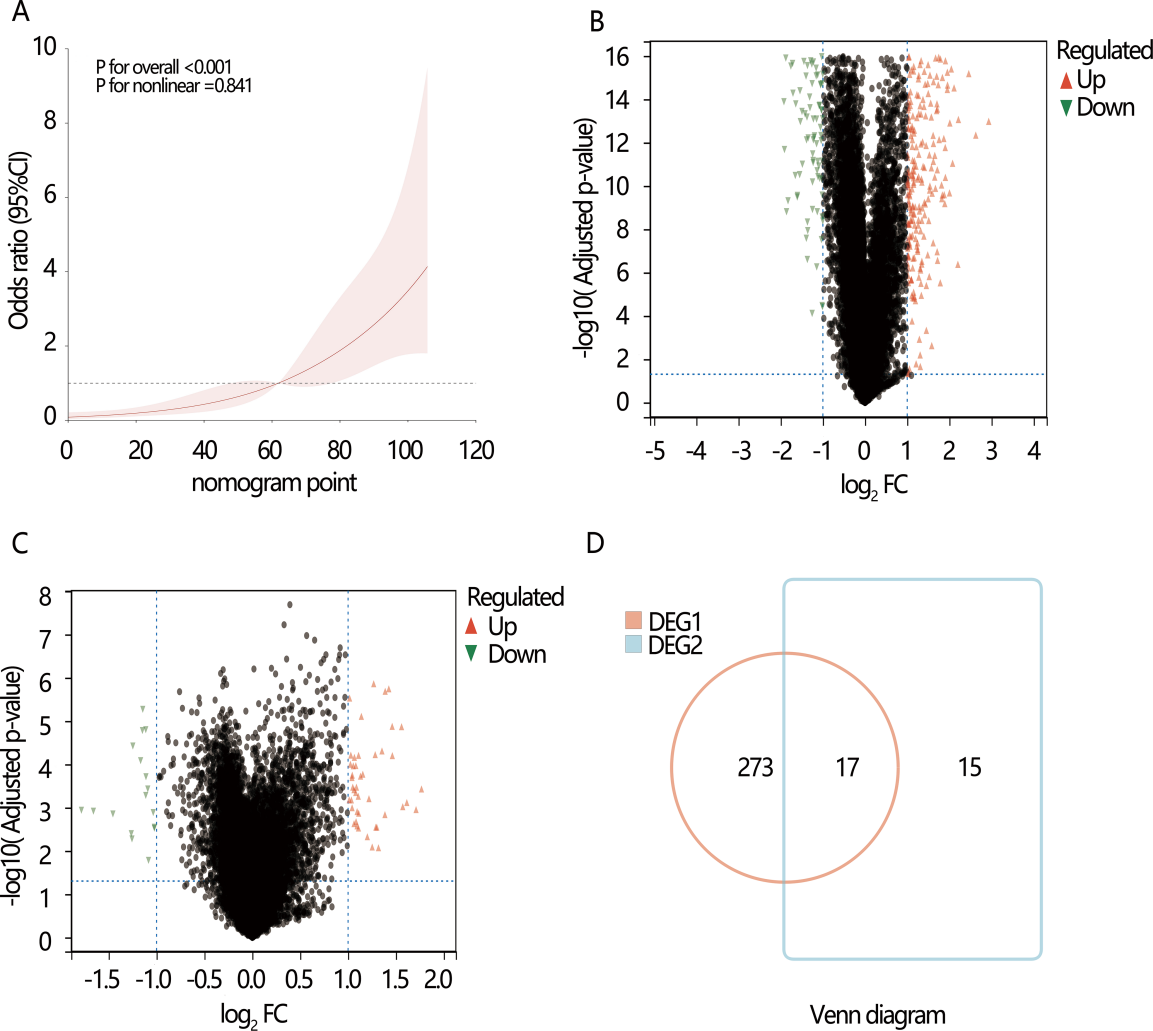
The potential genes and pathways exploration associated with the dedifferentiation. Volcano plots show the differentially expressed genes (DEGs). **(A)** The restricted cubic spline (RCS) analysis between nomogram point and dedifferentiation; **(B)** DGE1: DEGs between the high and low-risk dedifferentiation samples; **(C)** DGE2: DEGs between the high and low nomogram point samples; **(D)** Venn diagram shows the overlapping DEGs in DGE1 and DGE2.

**Figure 5:**
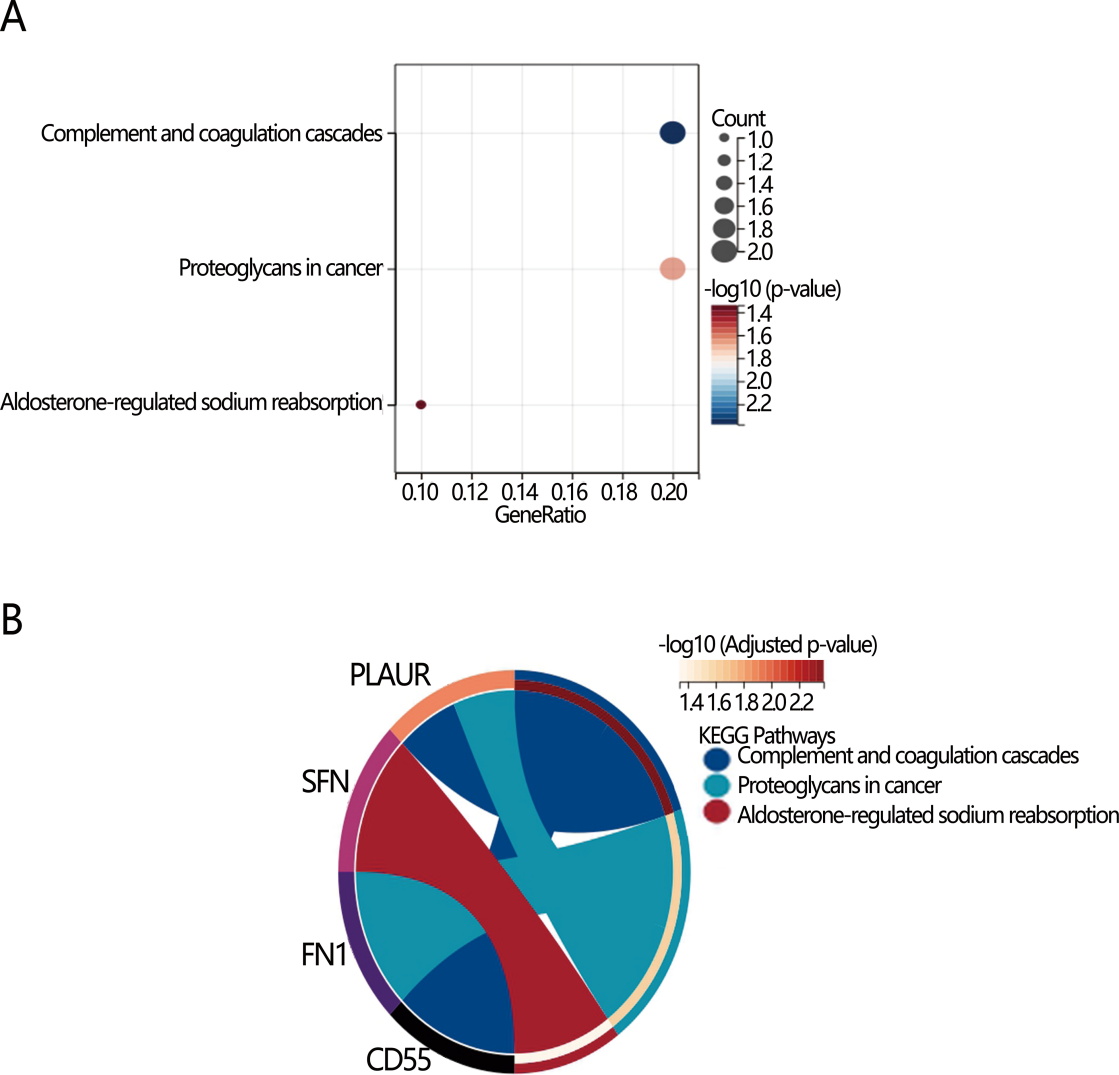
Visualization of KEGG enrichment analysis of 17 overlapping DEGs differential pathways. **(A)** Bubble diagram; **(B)** Chord diagram.

We next explored the potential mechanism associated with the PTC dedifferentiation. RCS analysis initially indicated a linear positive relationship between nomogram score and dedifferentiation risk (*P* for overall<0.001, *P* for nonlinear=0.841), suggesting the involvement of common biomarkers between them (**Figure 4A**). Therefore, we then explored the DEGs between high and low-risk dedifferentiation groups, as well as between high and low-nomogram score groups who were divided by their optimal cut-off value. According to the criteria of |log_2_ FC)|>1 and adjusted *P*-value<0.05 (**Figures 4B, C**), there were 290 DEGs obtained in the different dedifferentiation groups (**Supplementary Table S3**) and 32 DEGs obtained in the different nomogram score groups (**Supplementary Table S4**), with 17 overlapping genes between the two groups (**Supplementary Table S5**; **Figure 4D**). Among the 17 overlapping genes, we found 11 up-regulated genes and 6 down-regulated genes. KEGG enrichment analysis revealed three significant pathways: complement and coagulation cascades, proteoglycans in cancer, and aldosterone-regulated sodium reabsorption (all *P*<0.05) (**Figure 5A**). Among these, the complement and the coagulation cascades pathway were the main pathway, with *CD55* identified as a key factor (**Figure 5B**).

**Figure 6:**
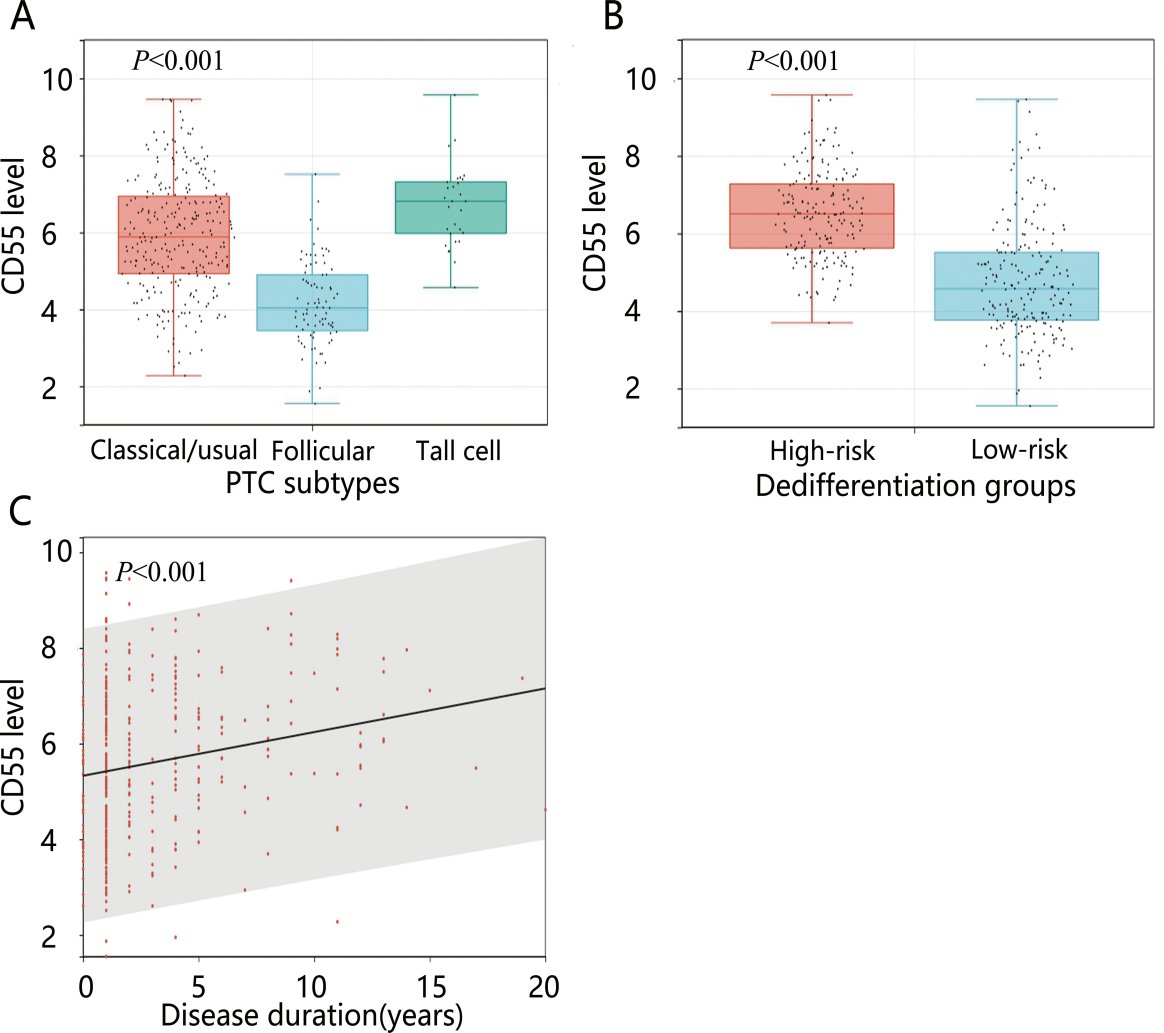
The expression levels of *CD55* in different variables. **(A)** PTC subtypes; **(B)** Dedifferentiation; **(C)** Disease duration.

We further analyzed the expression level of *CD55* between groups with different PTC subtypes, disease duration, and dedifferentiation (**Figures 6A–C**). The results showed significant upregulation of *CD55* expression in the high-risk dedifferentiation group. Tall cell PTC exhibited higher *CD55* expression levels compared to follicular PTC. Additionally, *CD55* expression levels demonstrated an increasing trend with prolonged disease duration.

## 4 Discussion

Clinical treatment response and prognosis in PTC patients can be evaluated using dedifferentiation, TMB, and immune scores. Our research utilized high-throughput sequencing data from the TCGA database to calculate TDS, TMB, and immune scores for PTC tumor samples. The influences of the three tumor biomarkers on PFI in PTC patients were further examined. Multivariable Cox analysis revealed that only TDS was an influence factor of PFI, highlighting the crucial role of cellular differentiation levels in patient prognosis. Therefore, investigating the high-risk clinical features and molecular mechanisms of high-risk dedifferentiated populations could facilitate early detection, enabling appropriate therapeutic interventions and potentially improving patient outcomes.

Multivariable logistic regression analysis of high-risk dedifferentiation indicated that PTC subtypes and disease duration were significant risk factors for dedifferentiation. The nomogram model combining PTC subtypes and disease duration demonstrated good discriminative ability and can be clinically beneficial. PTC exhibits various variant subtypes (34). The American Thyroid Association classifies PTC subtypes such as tall cell, diffuse sclerosing, and hobnail as intermediate risk based on their aggressiveness, with TCPTC being the most common aggressive PTC subtype. These subtypes are less differentiated than classic PTC (35–37). Our results also revealed that TCPTC had the highest risk of dedifferentiation compared with classic/usual PTC and follicular variant PTC. In KEGG pathway analysis, we found that *FN1* as a marker of epithelial-mesenchymal transition (EMT) was involved in PTC dedifferentiation. EMT plays a key role in PTC invasion and anaplastic transformation (38, 39). It has been suggested that as the disease progresses, some PTCs may undergo dedifferentiation, transforming into anaplastic thyroid cancer (ATC) and poorly differentiated thyroid cancer, accompanied by more aggressive pathological and clinical behaviors (8). A median disease duration transforming PTC into ATC has been reported as 6 years (40). Furthermore, in terms of molecular classification, PTC can be categorized into *BRAF*-like and *RAS*-like PTC (41). Different oncogenic drivers may cause PTC to exhibit different degrees of differentiation (or TDS) (42). TDS is a comprehensive indicator related to the expression and function of genes involved in iodine metabolism (43). Low TDS may cause patients to develop radioactive iodine resistance, leading to poor prognosis and high mortality (44).

Further, our study identified 17 genes associated with PTC dedifferentiation. Enrichment analysis of these DEGs revealed core pathways and hub genes, potentially offering new insights into dedifferentiation mechanisms. KEGG analysis demonstrated that the *PLAUR* gene was involved in the proteoglycans in cancer. Research has shown that the *PLAUR* gene plays a role in PTC differentiation and *HER2*-positive breast cancer metastasis (45, 46). Evidence suggests that the *PLAUR* gene activates the urokinase fibrinogen activator receptor (uPAR). uPAR promotes the activation of fibrinogen, which breaks down the peri-tumor stroma and basement membrane (e.g., fibronectin, proteoglycans), creating conditions for tumor invasion and metastasis (47).

Our analysis revealed that the most important pathways in the KEGG enrichment analysis were the complement and coagulation cascades. The complement system is crucial for eliminating foreign microorganisms and regulating both innate and adaptive immunity (48). Research has demonstrated that the coagulation and complement cascade pathway has multiple positive and negative effects on the incidence, progression, and prognosis of tumors, as well as influencing tumor microenvironment components (49–51). Activation of the complement pathway leads to the formation of membrane attack complexes that induce cellular activity under target cell lysis or shedding. Inappropriate complement activation or altered expression of complement regulatory proteins inhibits the elimination of tumor cells by immune cells, which is associated with a variety of tumors (52, 53) Coagulation begins after complement activation, subsequently triggering platelet activity (54). Activated platelets can influence immune cell function, leading to inflammation (55). The previous study has confirmed thrombocytosis accompanying inflammation-related colorectal cancer and highlighted the crucial role of interleukin-6 (IL-6) in this process (56). Research has shown that IL-6, activin-A, and granulocyte colony-stimulating factor (G-CSF) in the tumor microenvironment promote the dedifferentiation of hepatocellular carcinoma cells as well as thyroid cancer cells (57, 58). In the present study, *CD55* was identified as a key gene in the coagulation and complement cascade pathway. Known as a complement decay accelerator, *CD55* is involved in tumor dedifferentiation, proliferation, invasion, and migration and its upregulation may be associated with tumor progression (59–61). Previous studies have shown that the complement system is activated in PTC and regulated through *CD46*, *CD55*, and *CD59* (62). *CD55* protects thyroid cancer cells from complement-mediated attack and promotes carcinogenesis by allowing tumor cells to escape from cytolysis (63). The results of our analysis suggested that *CD55* expression in patients was up-regulated with increasing disease duration (or in TCPTC), potentially activating complement and coagulation cascade pathway, thus promoting cancer cell dedifferentiation. Based on these results we propose the following clinical recommendations: first, *CD55* can be used as a potential prognostic marker for identifying patients at high risk of dedifferentiation, and it is recommended that *CD55* immunohistochemical evaluation can be added to postoperative pathology testing and follow-up monitoring should be intensified (e.g., by shortening the interval between follow-ups or by increasing the number of imaging studies) for patients at high risk. Second, more aggressive treatment strategies should be considered for patients with high *CD55* expression, such as expanding the extent of surgery or consideration of adjuvant therapies targeting *CD55*-related pathways (e.g., complement signaling or EMT pathway). In addition, future studies should further validate the predictive value of *CD55* and explore its molecular mechanisms to develop possible targeted interventions, such as immunotherapy combined with complement-modulating therapies. Ultimately, the clinical application of *CD55* needs to be optimized through multicenter prospective studies to optimize detection criteria and integrate other molecular markers to improve the accuracy of risk stratification.

The innovation of this study is to combine clinical and genetic data to achieve individualized prognostic assessment of PTC patients, providing a more accurate tool for clinical practice and facilitating personalized treatment and management of PTC patients. Nevertheless, there are some limitations of this study: (1) No external validation was performed; (2) There were no clear boundaries for TDS subgroups. In this study, TDS was grouped by the optimal cut-off value, while some studies were grouped by median or 0 (43, 64). Therefore, future research should investigate optimal TDS grouping thresholds; (3) Data were only obtained from the TGCA database, which limited the extrapolation of results due to the influences of region and ethnicity.

## 5 Conclusion

In terms of clinical features, our findings revealed that disease duration and PTC subtypes were risk factors for high-risk dedifferentiation. At the molecular level, we identified 17 DEGs linked to high-risk dedifferentiation, potentially playing crucial roles in regulating PTC dedifferentiation. The complement coagulation cascade pathway may play a dominant role in PTC dedifferentiation, and the *CD55* could be the critical gene for PTC dedifferentiation, but further studies are needed to validate the results of these findings.

## Data Availability

The raw data supporting the conclusions of this article will be made available by the authors, without undue reservation.
