# Supplementary material for: Identification of risk factors for high-risk dedifferentiation in papillary thyroid carcinoma and construction of discriminative model
Source: Front Oncol. 2025 Jun 4;15:1535966. doi: 10.3389/fonc.2025.1535966 (PMC12174466; doi:10.3389/fonc.2025.1535966)
Supplement: Supplementary file 2 [file Table2.docx]

**Supplementary table 2.** Results of collinearity analysis for the 13 variables.

| **Variables** | **VIF** |
| --- | --- |
| Tumor length | 6.796 |
| Tumor width | 6.789 |
| Number of positive lymph nodes | 2.889 |
| Number of examined lymph nodes | 2.717 |
| T stage | 1.847 |
| Stage | 1.556 |
| N stage | 1.497 |
| PTC subtypes | 1.330 |
| Disease duration | 1.208 |
| Residual tumor | 1.196 |
| Follow-up after radiation treatment | 1.185 |
| Medical history of thyroid gland disorder | 1.098 |
| Race | 1.061 |

*Abbreviation: VIF, variance inflation factor; PTC, Papillary thyroid carcinoma.
