# Supplementary material for: Identification of risk factors for high-risk dedifferentiation in papillary thyroid carcinoma and construction of discriminative model
Source: Front Oncol. 2025 Jun 4;15:1535966. doi: 10.3389/fonc.2025.1535966 (PMC12174466; doi:10.3389/fonc.2025.1535966)
Supplement: Supplementary file 3 [file Table3.docx]

**Supplementary table 3.** 290 DEGs between high-risk dedifferentiation group and the low-risk differentiation group (DEG1).

| **Genes** | **log_2_FC** | **\|log_2_FC\|** | ***P*-value** |
| --- | --- | --- | --- |
| TPO | -4.727 | 4.727 | <0.001 |
| HGD | -2.505 | 2.505 | <0.001 |
| DIO1 | -4.065 | 4.065 | <0.001 |
| SLC4A4 | -1.988 | 1.988 | <0.001 |
| MT1G | -4.846 | 4.846 | <0.001 |
| CDON | -1.398 | 1.398 | <0.001 |
| TFF3 | -3.910 | 3.910 | <0.001 |
| FAM167A | -2.661 | 2.661 | <0.001 |
| GNA14 | -1.614 | 1.614 | <0.001 |
| MT1F | -2.979 | 2.979 | <0.001 |
| NECTIN4 | 2.059 | 2.059 | <0.001 |
| LGI3 | -1.896 | 1.896 | <0.001 |
| SLC26A4-AS1 | -3.758 | 3.758 | <0.001 |
| SLC26A4 | -2.936 | 2.936 | <0.001 |
| SLC26A7 | -2.634 | 2.634 | <0.001 |
| AF131216.3 | -1.565 | 1.565 | <0.001 |
| MPPED2 | -1.422 | 1.422 | <0.001 |
| SORBS2 | -1.638 | 1.638 | <0.001 |
| FHL1 | -2.056 | 2.056 | <0.001 |
| SLC5A8 | -2.301 | 2.301 | <0.001 |
| DCSTAMP | 4.003 | 4.003 | <0.001 |
| OTOS | -2.679 | 2.679 | <0.001 |
| ITPR1 | -1.482 | 1.482 | <0.001 |
| ELMO1 | -1.616 | 1.616 | <0.001 |
| MACC1 | 1.227 | 1.227 | <0.001 |
| WSCD2 | -1.779 | 1.779 | <0.001 |
| ERBB3 | 1.586 | 1.586 | <0.001 |
| FN1 | 4.056 | 4.056 | <0.001 |
| BTBD11 | -1.406 | 1.406 | <0.001 |
| ARHGAP24 | -1.284 | 1.284 | <0.001 |
| CYP2S1 | 1.849 | 1.849 | <0.001 |
| KCNIP3 | -1.257 | 1.257 | <0.001 |
| TACSTD2 | 3.813 | 3.813 | <0.001 |
| PDLIM4 | 2.606 | 2.606 | <0.001 |
| IQGAP2 | -1.595 | 1.595 | <0.001 |
| B3GNT3 | 2.887 | 2.887 | <0.001 |
| C16orf89 | -2.144 | 2.144 | <0.001 |
| ITGA3 | 1.275 | 1.275 | <0.001 |
| HLF | -1.501 | 1.501 | <0.001 |
| TMEM79 | 1.152 | 1.152 | <0.001 |
| GJB3 | 1.838 | 1.838 | <0.001 |
| TCF7L1 | -1.296 | 1.296 | <0.001 |
| BID | 1.033 | 1.033 | <0.001 |
| PLCD3 | 1.812 | 1.812 | <0.001 |
| SPTBN2 | 1.102 | 1.102 | <0.001 |
| MRO | -1.034 | 1.034 | <0.001 |
| TBC1D2 | 1.382 | 1.382 | <0.001 |
| LRP2 | -1.585 | 1.585 | <0.001 |
| LAD1 | 1.329 | 1.329 | <0.001 |
| ELF3 | 1.603 | 1.603 | <0.001 |
| SULT1A2 | -1.137 | 1.137 | <0.001 |
| LDHD | -1.370 | 1.370 | <0.001 |
| GRB7 | 1.225 | 1.225 | <0.001 |
| SYT12 | 2.230 | 2.230 | <0.001 |
| EPHB3 | 1.390 | 1.390 | <0.001 |
| RPS29P11 | 2.184 | 2.184 | <0.001 |
| GRIN2C | -1.544 | 1.544 | <0.001 |
| SPX | -1.577 | 1.577 | <0.001 |
| GALNT17 | -1.380 | 1.380 | <0.001 |
| LINC01936 | -1.013 | 1.013 | <0.001 |
| PERP | 1.322 | 1.322 | <0.001 |
| KCNN4 | 2.441 | 2.441 | <0.001 |
| ESAM | -1.053 | 1.053 | <0.001 |
| KCNAB1 | -1.977 | 1.977 | <0.001 |
| COL23A1 | -1.970 | 1.970 | <0.001 |
| RUNX1 | 1.186 | 1.186 | <0.001 |
| CGNL1 | -1.457 | 1.457 | <0.001 |
| GPAM | -1.165 | 1.165 | <0.001 |
| AC062015.1 | -1.675 | 1.675 | <0.001 |
| PLEKHA6 | 1.054 | 1.054 | <0.001 |
| DEPTOR | -1.410 | 1.410 | <0.001 |
| SOD3 | -1.981 | 1.981 | <0.001 |
| IRS1 | -1.184 | 1.184 | <0.001 |
| PTPRE | 1.445 | 1.445 | <0.001 |
| KCNQ3 | 1.246 | 1.246 | <0.001 |
| 2-Mar | -1.023 | 1.023 | <0.001 |
| AQP4 | -1.568 | 1.568 | <0.001 |
| PROS1 | 1.962 | 1.962 | <0.001 |
| GALNT10 | -1.071 | 1.071 | <0.001 |
| DSC2 | 1.075 | 1.075 | <0.001 |
| EVA1A | 1.658 | 1.658 | <0.001 |
| TMPRSS4 | 2.065 | 2.065 | <0.001 |
| ZMAT4 | -1.531 | 1.531 | <0.001 |
| CYP1B1 | 2.295 | 2.295 | <0.001 |
| MT1H | -3.333 | 3.333 | <0.001 |
| IGFBPL1 | -1.309 | 1.309 | <0.001 |
| FSTL3 | 1.397 | 1.397 | <0.001 |
| TFCP2L1 | -1.569 | 1.569 | <0.001 |
| CEBPA-DT | -1.105 | 1.105 | <0.001 |
| SLC34A2 | 3.860 | 3.860 | <0.001 |
| S100A6 | 1.509 | 1.509 | <0.001 |
| MMP15 | -1.191 | 1.191 | <0.001 |
| HMGA1 | 1.186 | 1.186 | <0.001 |
| KRT19 | 2.650 | 2.650 | <0.001 |
| SNTA1 | -1.103 | 1.103 | <0.001 |
| TGFBR1 | 1.182 | 1.182 | <0.001 |
| NGEF | 1.802 | 1.802 | <0.001 |
| SOX4 | 1.231 | 1.231 | <0.001 |
| MATN2 | -1.519 | 1.519 | <0.001 |
| EBF4 | -1.805 | 1.805 | <0.001 |
| CYP4F32P | -1.617 | 1.617 | <0.001 |
| C19orf33 | 2.218 | 2.218 | <0.001 |
| FCGBP | -1.536 | 1.536 | <0.001 |
| SERPINA1 | 3.084 | 3.084 | <0.001 |
| LY6E | 1.629 | 1.629 | <0.001 |
| GABRB2 | 2.086 | 2.086 | <0.001 |
| KLK7 | 2.668 | 2.668 | <0.001 |
| QSOX1 | 1.084 | 1.084 | <0.001 |
| ISM1 | -1.505 | 1.505 | <0.001 |
| LAMB3 | 2.554 | 2.554 | <0.001 |
| CD55 | 1.922 | 1.922 | <0.001 |
| RSPO4 | 1.653 | 1.653 | <0.001 |
| AC002401.4 | 1.316 | 1.316 | <0.001 |
| ANXA2 | 1.400 | 1.400 | <0.001 |
| PDE8B | -1.513 | 1.513 | <0.001 |
| PTPRU | 1.274 | 1.274 | <0.001 |
| CLCNKA | -1.325 | 1.325 | <0.001 |
| MIOX | -1.387 | 1.387 | <0.001 |
| AL031710.1 | -1.526 | 1.526 | <0.001 |
| ETHE1 | 1.106 | 1.106 | <0.001 |
| TMPRSS6 | 2.483 | 2.483 | <0.001 |
| SELENOV | -1.256 | 1.256 | <0.001 |
| ABTB2 | 1.165 | 1.165 | <0.001 |
| PGF | -1.580 | 1.580 | <0.001 |
| KLK10 | 2.661 | 2.661 | <0.001 |
| MUC21 | 1.796 | 1.796 | <0.001 |
| MET | 1.651 | 1.651 | <0.001 |
| ALDH3B1 | 1.192 | 1.192 | <0.001 |
| TMEM171 | -1.257 | 1.257 | <0.001 |
| CAPSL | -1.332 | 1.332 | <0.001 |
| BCL2 | -1.054 | 1.054 | <0.001 |
| SFN | 2.391 | 2.391 | <0.001 |
| DIO2 | -1.587 | 1.587 | <0.001 |
| LGALS3 | 1.944 | 1.944 | <0.001 |
| MT1M | -1.575 | 1.575 | <0.001 |
| SEMA3D | -1.780 | 1.780 | <0.001 |
| DOK7 | 1.268 | 1.268 | <0.001 |
| SPOCK2 | 2.332 | 2.332 | <0.001 |
| MAMLD1 | 1.122 | 1.122 | <0.001 |
| PKHD1L1 | -1.678 | 1.678 | <0.001 |
| KRT80 | 1.471 | 1.471 | <0.001 |
| PPARGC1A | -1.180 | 1.180 | <0.001 |
| C11orf74 | -1.040 | 1.040 | <0.001 |
| GGCT | 1.082 | 1.082 | <0.001 |
| AC091563.1 | -1.147 | 1.147 | <0.001 |
| TMEM178B | -1.266 | 1.266 | <0.001 |
| CTSH | 1.521 | 1.521 | <0.001 |
| LINC02568 | -1.821 | 1.821 | <0.001 |
| LINC01789 | -1.973 | 1.973 | <0.001 |
| FAM20C | 1.014 | 1.014 | <0.001 |
| ID4 | -1.123 | 1.123 | <0.001 |
| CST6 | 3.017 | 3.017 | <0.001 |
| LY6G6C | 1.015 | 1.015 | <0.001 |
| OR2W3 | -1.010 | 1.010 | <0.001 |
| NXN | 1.080 | 1.080 | <0.001 |
| PALM | 1.346 | 1.346 | <0.001 |
| SFTPB | 3.748 | 3.748 | <0.001 |
| MUC1 | 2.119 | 2.119 | <0.001 |
| AC254633.1 | 2.283 | 2.283 | <0.001 |
| WDR72 | -1.264 | 1.264 | <0.001 |
| LRRC2 | -1.030 | 1.030 | <0.001 |
| FAM155B | -1.752 | 1.752 | <0.001 |
| LMOD1 | -1.225 | 1.225 | <0.001 |
| RCAN2 | -1.079 | 1.079 | <0.001 |
| ST6GALNAC5 | 1.533 | 1.533 | <0.001 |
| AHNAK2 | 1.464 | 1.464 | <0.001 |
| MHENCR | -1.025 | 1.025 | <0.001 |
| MT1DP | -1.022 | 1.022 | <0.001 |
| SEL1L3 | 1.342 | 1.342 | <0.001 |
| SDC4 | 1.552 | 1.552 | <0.001 |
| ITGB8 | 1.006 | 1.006 | <0.001 |
| S100A11 | 1.044 | 1.044 | <0.001 |
| C16orf74 | -1.027 | 1.027 | <0.001 |
| TMEM92 | 1.035 | 1.035 | <0.001 |
| MXRA8 | 1.747 | 1.747 | <0.001 |
| PDE5A | 1.673 | 1.673 | <0.001 |
| CDH16 | -1.879 | 1.879 | <0.001 |
| SLC25A47P1 | 1.787 | 1.787 | <0.001 |
| TNFRSF21 | 1.330 | 1.330 | <0.001 |
| DUSP5 | 1.906 | 1.906 | <0.001 |
| RGS16 | -1.288 | 1.288 | <0.001 |
| AL157871.6 | -1.450 | 1.450 | <0.001 |
| SLC16A2 | -1.225 | 1.225 | <0.001 |
| CA4 | -1.719 | 1.719 | <0.001 |
| SDF2L1 | -1.262 | 1.262 | <0.001 |
| PPL | 1.303 | 1.303 | <0.001 |
| TNFRSF12A | 1.495 | 1.495 | <0.001 |
| FAM240C | -1.366 | 1.366 | <0.001 |
| DUOXA2 | -1.759 | 1.759 | <0.001 |
| CDC42EP5 | 1.154 | 1.154 | <0.001 |
| SLC25A15 | -1.159 | 1.159 | <0.001 |
| CXCL17 | 2.046 | 2.046 | <0.001 |
| NRIP1 | 1.096 | 1.096 | <0.001 |
| AHR | 1.149 | 1.149 | <0.001 |
| WARS1P1 | 1.404 | 1.404 | <0.001 |
| TIMP1 | 1.952 | 1.952 | <0.001 |
| LCN2 | 2.459 | 2.459 | <0.001 |
| BTNL9 | -1.003 | 1.003 | <0.001 |
| IL1RAP | 1.058 | 1.058 | <0.001 |
| GSTM3 | -1.125 | 1.125 | <0.001 |
| ANXA1 | 1.539 | 1.539 | <0.001 |
| MFGE8 | 1.274 | 1.274 | <0.001 |
| WNT4 | -1.158 | 1.158 | <0.001 |
| LIPG | -1.399 | 1.399 | <0.001 |
| MGAT3 | 1.698 | 1.698 | <0.001 |
| CAMK2N1 | 1.834 | 1.834 | <0.001 |
| WNT10A | 1.264 | 1.264 | <0.001 |
| SYTL5 | 1.623 | 1.623 | <0.001 |
| ICAM1 | 1.486 | 1.486 | <0.001 |
| ATP6V0E2 | -1.014 | 1.014 | <0.001 |
| CWH43 | -1.255 | 1.255 | <0.001 |
| PLA2R1 | -1.050 | 1.050 | <0.001 |
| CTXN1 | 1.425 | 1.425 | <0.001 |
| CLDN10 | 1.735 | 1.735 | <0.001 |
| TRIM58 | -1.054 | 1.054 | <0.001 |
| ALOX15B | 1.956 | 1.956 | <0.001 |
| ALOX5 | 2.111 | 2.111 | <0.001 |
| DTX4 | 1.951 | 1.951 | <0.001 |
| RP11-69E11.4 | -1.543 | 1.543 | <0.001 |
| UPP1 | 1.077 | 1.077 | <0.001 |
| TG | -1.709 | 1.709 | <0.001 |
| ITGA2 | 1.499 | 1.499 | <0.001 |
| RASD2 | 1.230 | 1.230 | <0.001 |
| ANXA2P2 | 1.035 | 1.035 | <0.001 |
| ANXA3 | 1.266 | 1.266 | <0.001 |
| MT1X | -1.314 | 1.314 | <0.001 |
| BASP1 | 1.373 | 1.373 | <0.001 |
| RND3 | 1.084 | 1.084 | <0.001 |
| LINC01886 | -1.923 | 1.923 | <0.001 |
| CLIP3 | 1.021 | 1.021 | <0.001 |
| CDH3 | 1.557 | 1.557 | <0.001 |
| COL8A2 | 1.705 | 1.705 | <0.001 |
| BMP8A | -1.643 | 1.643 | <0.001 |
| CLCNKB | -1.079 | 1.079 | <0.001 |
| PRICKLE1 | 1.073 | 1.073 | <0.001 |
| STRA6 | 1.305 | 1.305 | <0.001 |
| TMEM88 | -1.089 | 1.089 | <0.001 |
| C1orf116 | 1.397 | 1.397 | <0.001 |
| TMSB10 | 1.038 | 1.038 | <0.001 |
| TSPAN33 | -1.306 | 1.306 | <0.001 |
| S100A10 | 1.714 | 1.714 | <0.001 |
| BRINP1 | 1.022 | 1.022 | <0.001 |
| GPER1 | -1.032 | 1.032 | <0.001 |
| SLC25A29 | -1.034 | 1.034 | <0.001 |
| LIPH | 1.697 | 1.697 | <0.001 |
| KIT | -1.495 | 1.495 | <0.001 |
| IL1RN | 1.274 | 1.274 | <0.001 |
| MIR621 | -1.424 | 1.424 | <0.001 |
| ITGB4 | 1.326 | 1.326 | <0.001 |
| CTSC | 1.437 | 1.437 | <0.001 |
| SCD | 1.134 | 1.134 | <0.001 |
| EBI3 | 1.030 | 1.030 | <0.001 |
| TM4SF1 | 1.571 | 1.571 | <0.001 |
| OGDHL | -1.377 | 1.377 | <0.001 |
| ARNTL | 1.228 | 1.228 | <0.001 |
| VEGFA | -1.007 | 1.007 | <0.001 |
| KIAA1324 | -1.225 | 1.225 | <0.001 |
| IPCEF1 | -1.150 | 1.150 | <0.001 |
| SLC27A6 | 2.213 | 2.213 | <0.001 |
| AP002358.1 | 1.039 | 1.039 | <0.001 |
| CHI3L1 | 2.933 | 2.933 | <0.001 |
| MT1E | -1.534 | 1.534 | <0.001 |
| SLC22A31 | 2.010 | 2.010 | <0.001 |
| VTCN1 | 1.117 | 1.117 | <0.001 |
| VAV3 | -1.057 | 1.057 | <0.001 |
| DEPDC1B | 1.023 | 1.023 | <0.001 |
| MAP3K5 | 1.023 | 1.023 | <0.001 |
| MPZL2 | 1.084 | 1.084 | <0.001 |
| PPP1R1B | 1.120 | 1.120 | <0.001 |
| LDLR | 1.169 | 1.169 | <0.001 |
| LINC02560 | 1.158 | 1.158 | <0.001 |
| SLPI | 2.625 | 2.625 | <0.001 |
| SCEL | 1.614 | 1.614 | <0.001 |
| EXOC3L2 | -1.045 | 1.045 | <0.001 |
| FAM43A | 1.086 | 1.086 | <0.001 |
| PRR15L | -1.198 | 1.198 | <0.001 |
| ALDH1A1 | -1.214 | 1.214 | <0.001 |
| RAP1GAP | -1.349 | 1.349 | <0.001 |
| PLAU | 1.868 | 1.868 | <0.001 |
| S100A4 | 1.757 | 1.757 | <0.001 |
| GALE | 1.123 | 1.123 | <0.001 |
| SLC1A1 | -1.226 | 1.226 | <0.001 |
| TNFRSF11B | -1.388 | 1.388 | <0.001 |
| PLVAP | -1.040 | 1.040 | <0.001 |
| CCL13 | 1.365 | 1.365 | <0.001 |
| SCARA3 | 1.622 | 1.622 | <0.001 |
| ADM2 | -1.149 | 1.149 | <0.001 |
| PLAUR | 1.325 | 1.325 | <0.001 |
| NFE2L3 | 1.448 | 1.448 | <0.001 |
| BHLHE40 | 1.118 | 1.118 | <0.001 |
