# Supplementary material for: Identification of risk factors for high-risk dedifferentiation in papillary thyroid carcinoma and construction of discriminative model
Source: Front Oncol. 2025 Jun 4;15:1535966. doi: 10.3389/fonc.2025.1535966 (PMC12174466; doi:10.3389/fonc.2025.1535966)
Supplement: Supplementary file 4 [file Table4.docx]

**Supplementary table 4.** 32 DEGs between high and low nomogram score groups (DEG2).

| **Genes** | **log_2_FC** | **\|log_2_FC\|** | ***P*-value** |
| --- | --- | --- | --- |
| S100A9 | 1.268 | 1.268 | <0.001 |
| MMP7 | 1.425 | 1.425 | <0.001 |
| IL1RL1 | 1.387 | 1.387 | <0.001 |
| PLAUR | 1.016 | 1.016 | <0.001 |
| FAM155B | -1.145 | 1.145 | <0.001 |
| MARCO | 1.139 | 1.139 | <0.001 |
| LYZ | 1.461 | 1.461 | <0.001 |
| KRT19 | 1.559 | 1.559 | <0.001 |
| DUOXA2 | -1.113 | 1.113 | <0.001 |
| C16orf89 | -1.148 | 1.148 | <0.001 |
| FAM167A | -1.248 | 1.248 | <0.001 |
| SFN | 1.354 | 1.354 | <0.001 |
| ALOX5 | 1.283 | 1.283 | <0.001 |
| FGFBP1 | 1.029 | 1.029 | <0.001 |
| CTHRC1 | 1.109 | 1.109 | <0.001 |
| LCN2 | 1.462 | 1.462 | <0.001 |
| S100A10 | 1.066 | 1.066 | <0.001 |
| HHATL | -1.167 | 1.167 | <0.001 |
| CD55 | 1.025 | 1.025 | <0.001 |
| DUSP5 | 1.081 | 1.081 | <0.001 |
| CCL17 | 1.054 | 1.054 | <0.001 |
| MUC1 | 1.154 | 1.154 | <0.001 |
| GPNMB | 1.044 | 1.044 | <0.001 |
| SPP1 | 1.130 | 1.130 | <0.001 |
| SFTPB | 2.020 | 2.020 | <0.001 |
| LINC01886 | -1.111 | 1.111 | <0.001 |
| CEACAM6 | 1.041 | 1.041 | <0.001 |
| TNC | 1.049 | 1.049 | <0.001 |
| HLA-DQA2 | 1.087 | 1.087 | <0.001 |
| FN1 | 1.767 | 1.767 | <0.001 |
| SLC26A7 | -1.092 | 1.092 | <0.001 |
| COL3A1 | 1.062 | 1.062 | <0.001 |
