# Supplementary material for: Identification of risk factors for high-risk dedifferentiation in papillary thyroid carcinoma and construction of discriminative model
Source: Front Oncol. 2025 Jun 4;15:1535966. doi: 10.3389/fonc.2025.1535966 (PMC12174466; doi:10.3389/fonc.2025.1535966)
Supplement: Supplementary file 5 [file Table5.docx]

**Supplementary table 5.** 17 overlapping genes between DEG1 and DEG2.

| **Overlapping genes** | **Expression** |
| --- | --- |
| FAM167A | Down-regulated |
| SLC26A7 | Down-regulated |
| FN1 | Up-regulated |
| C16orf89 | Down-regulated |
| KRT19 | Up-regulated |
| CD55 | Up-regulated |
| SFN | Up-regulated |
| SFTPB | Up-regulated |
| MUC1 | Up-regulated |
| FAM155B | Down-regulated |
| DUSP5 | Up-regulated |
| DUOXA2 | Down-regulated |
| LCN2 | Up-regulated |
| ALOX5 | Up-regulated |
| LINC01886 | Down-regulated |
| S100A10 | Up-regulated |
| PLAUR | Up-regulated |
